# An Active-Matrix Organic Light-Emitting Diode Pixel Circuit Featuring Mobility Compensation for Portable Applications

**DOI:** 10.3390/mi14091785

**Published:** 2023-09-18

**Authors:** Ching-Lin Fan, Wei-Yu Lin, Shih-Yang Liu

**Affiliations:** 1 Graduate Institute of Electro-Optical Engineering, National Taiwan University of Science and Technology, No. 43, Sec. 4, Keelung Rd., Da’an Dist., Taipei City 106, Taiwan; d11019001@mail.ntust.edu.tw; 2 Department of Electronic and Computer Engineering, National Taiwan University of Science and Technology, No. 43, Sec. 4, Keelung Rd., Da’an Dist., Taipei City 106, Taiwan; d11002006@mail.ntust.edu.tw

**Keywords:** low-temperature polycrystalline oxide (LTPO), active-matrix organic light-emitting diode (AMOLED), mobility compensating

## Abstract

A 6T1C pixel circuit based on low-temperature polycrystalline oxide (LTPO) technology for portable active-matrix organic light-emitting diode (AMOLED) display applications is proposed in this paper. For superior high-end portable applications including 4K high resolution and high PPI (pixels per inch), the proposed pixel circuit employs a single storage capacitor and signal sharing switch-control design and provides low-voltage driving and immunity to the IR-drop issue and OLED degradation. Furthermore, the threshold voltage and mobility-compensating capabilities are improved by both compensation mechanisms, which are based on a negative feedback system, and mobility-related compensation parameters. Simulation results reveal that threshold voltage variations of ±0.33 V in the driving thin-film transistors can be well sensed and compensated while the maximum OLED current shift is 4.25%. The maximum variation in OLED currents within all gray levels is only 1.05% with mobility variations of ±30%. As a result, the proposed 6T1C pixel circuit is a good candidate for portable AMOLED display usage.

## 1. Introduction

Active-matrix organic light-emitting diodes (AMOLEDs) have received increasing interest in display applications due to their versatility of colors, fast response time, high contrast ratio, and low power consumption [1,2,3]. To improve the immersive display experience, both the high resolution and high pixels per inch (PPI) features are essential in high-end portable displays. Power dissipation is also an important consideration for power-efficient AMOLED displays. Recently, AMOLED displays incorporating LTPO (low-temperature polycrystalline oxide) technologies have attracted much attention because it combines the advantages of low-temperature poly-silicon thin-film transistors (LTPS TFTs) and metal-oxide-semiconductor TFTs (oxide TFTs) by using both TFTs in the pixel circuit. Due to a high field-effect mobility and outstanding current-driving capability, LTPS TFTs have been widely used in mobile applications to achieve low-power driving [4,5,6,7]. However, the non-uniform LTPS TFT characteristics, such as the threshold voltage (V_TH_) shift and carrier mobility variations, are serious consequences of the crystallization process [8,9]. Furthermore, the LTPS TFT generally suffers from a high leakage current due to its polycrystalline structure [10,11]. In contrast to LTPS TFTs, oxide TFTs feature superior uniformity and an extremely low leakage current [12,13]. Therefore, the applications of oxide TFTs are primitively focused on the large-sized AMOLED panel or the smartwatch with low frame rate support [14,15,16,17]. Driving with LTPS TFT technologies is indispensable for power saving in portable AMOLED displays. To achieve high-performance image quality, the outstanding current-driving capability of LTPS technologies can improve the programming process efficiency. In addition, based on the advantage of a low leakage current, using oxide TFTs as a switch can suppress data loss from the storage capacitor. However, as mentioned above, both the variations in threshold voltage and mobility will lead to significant non-uniform OLED current levels and causes serious image color distortion [18,19].

As a result, various compensating pixel circuits based on the voltage-programming scheme have been proposed to resolve the driving TFT non-uniformity issues. Lai et al. [20] proposed a 4T2C pixel circuit that was used in an 8K4K resolution, large-sized AMOLED panel that effectively compensated for both the threshold voltage and mobility variations as well as the IR-drop issues. Nevertheless, the screen resolution and bezel area may be limited by the control signal line complexity and two storage capacitors in portable applications. Park et al. [21] proposed a 3T1C pixel circuit to compensate for threshold voltage and mobility variations using a capacitor-coupling mechanism between the storage capacitor and natural OLED capacitance (C_OLED_). This 3T1C pixel circuit may be beneficial to high-PPI portable applications with a simple structure. However, the intrinsic OLED capacitor is voltage-dependent, indicating that the C_OLED_ value may vary with the change in bias voltage [22,23]. The non-uniform OLED driving current problems may be caused by the COLED fluctuations. A 7T1C pixel circuit proposed by Fu et al. [24] alleviated the variations in threshold voltage and mobility by operating with a feedback scheme. Conversely, the IR-drop issue and complicated control signal line design may cause a decrease in picture quality.

In this paper, a 6T1C LTPO pixel circuit with a novel operation method is proposed that can compensate for both the threshold voltage and field-effect mobility variations, being mainly for use in 4K (3840 × 2160, UHD) portable displays. The mobility compensation process of the proposed circuit is based on two different mechanisms that successfully minimize the influence of field-effect mobility variations. To improve the image quality, while increasing the screen resolution and PPI, the number of control signal lines and TFTs in the proposed circuit are simplified. The immunity to both IR-drop issues and the variations in V_TH_ of OLED (V_TH_OLED_) are favorable for presenting a uniform image. The proposed pixel circuit can produce a highly uniform and stable OLED current with 5 V low-voltage drive, operating with maximum error rates of 4.25% and 1.05% for OLED currents under the condition of V_TH_ variations of driving TFT at ±0.33 V and mobility variations of driving TFT at ±30%, respectively.

## 2. Proposed Pixel Circuit Operation

**Figure 1:**
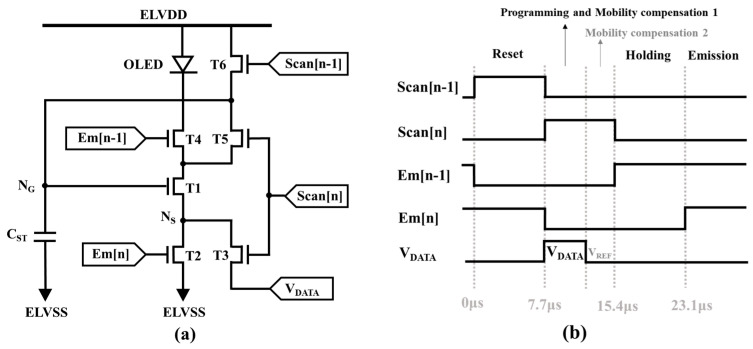
(**a**) The schematic of the proposed 6T1C pixel circuit and (**b**) the timing diagram of the control signal.

**Figure 2:**
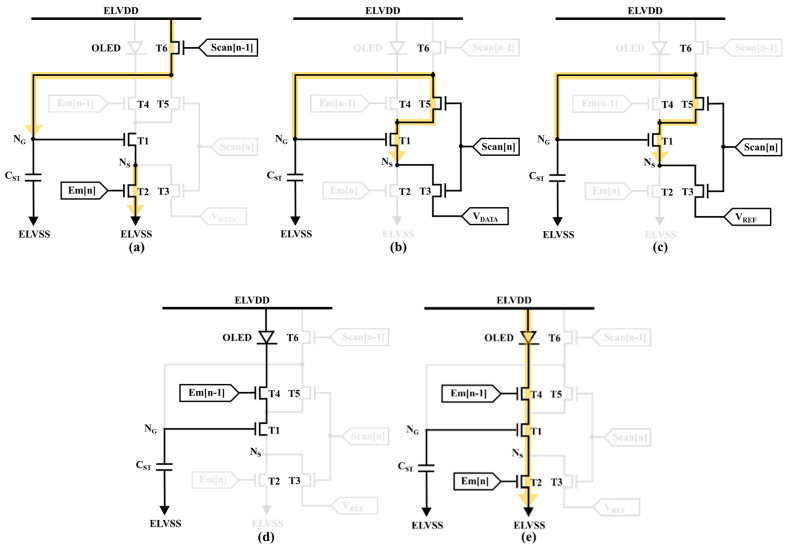
Schematic of pixel circuit operation, including (**a**) reset, (**b**) programming and first mobility compensation, (**c**) second mobility compensation, (**d**) holding, and (**e**) emission stages.

Figure 1a shows the circuit structure for the 6T1C LTPO pixel circuit, including an LTPS driving TFT (T1), two LTPS switching TFTs (T2 and T4), three oxide switching TFTs (T3, T5, and T6), one storage capacitor (C_ST_), and an OLED with top-anode structure. In addition, nodes N_G_ and N_S_ denote the gate (V_G_) and source (V_S_) voltages for T1, with the ability to control the pixel currents independently of the OLED operations. Figure 1b plots the corresponding timing diagram. To minimize the number of control signal lines, both Scan[n] and Scan[n − 1] (Em[n] and Em[n − 1]) are designed with the same pulse width. Herein the data voltage input scan period is set to 7.7 μs to accommodate the 4K resolution display. The proposed circuit is divided into five stages, as shown in Figure 2, presented in detail as follows.

### 2.1. Reset Stage

The scan signals of Scan[n − 1] and Em[n] are high to turn on T6 and T2, respectively. Scan[n] and Em[n − 1] are low to turn off T3, T5, and T4. The purpose of this stage is to refresh the V_GS_ of the driving TFT. The ELVDD is applied to N_G_ through T6 while the node N_S_ is discharged to ELVSS. Because of the open-circuit T4, the OLED is completely turned off to prevent image flicker.

### 2.2. Programming and First Mobility Compensation Stage

The scan signals for Scan[n − 1] and Em[n] are low to turn off T6 and T2. Em[n − 1] remains low to prevent the current flowing through the OLED. The objective of this stage is to compensate for V_TH_ variations of T1 while inputting data voltage signals simultaneously, and also to effectively alleviate the mobility variation in T1. Scan[n] goes high to turn on T3 and T5, and a data voltage is applied to node N_S_ via T3. The diode-connected structure, consisting of T1, T3, and T5, discharges node N_G_ from ELVDD to V_DATA_ + V_TH_T1_. Therefore, the voltage V_DATA_ + V_TH_T1_ is stored in C_ST_ at the end of the programming stage. Moreover, the first mobility compensation mechanism is based on a negative feedback system as the field-effect mobility of T1 increases. Increasing discharge current will drift from node N_G_ to node N_S_ as well as cause the voltage level of node N_G_ to decrease. As a result, the driving TFT gate voltage will drop discharge and reduce the discharge current of the field-effect mobility fluctuation, which is irrelevant to the discharge current.

### 2.3. Second Mobility Compensation Stage

The scan signals for Scan[n − 1], Em[n], and Em[n − 1] remain low to turn off T6, T2, and T4, respectively. Scan[n] remains high to turn on T3 and T5, and a low voltage of V_REF_ is applied to node N_S_ via T3. The voltage level of node N_G_ will be further discharged from V_DATA_ + V_TH_T1_ to V_DATA_ + V_TH_T1_ − ΔV_μ_T1_ within a period of 0.3 μs. Herein the ΔV_μ_T1_ value is proportional to the field-effect mobility of T1. Therefore, the mobility-related voltage of V_DATA_ + V_TH_T1_ − ΔV_μ_T1_ is stored in C_ST_ after the second mobility compensation stage.

### 2.4. Holding Stage

The scan signal for Scan[n] goes low to turn off T3 and T5. Scan[n − 1] and Em[n] remain low to turn off T6 and T2. Because of the floating node N_S_, the driving TFT of T1 is turned off. As a result, even though Em[n − 1] turns on T4, there is no driving current flowing through the OLED. Furthermore, the stored charges for C_ST_ are held well due to the low leakage current application of oxide TFTs including T5 and T6.

### 2.5. Emission Stage

The scan signals for Scan[n] and Scan[n − 1] remain low to turn off T3, T5, and T6, and Em[n − 1] remains high. The gate voltage (N_G_) is sustained at V_DATA_ + V_TH_T1_ − ΔV_μ_T1_ and the ELVSS is applied to the source voltage (N_S_) while Em[n] goes high to turn on T2. Because the driving TFT operates in the saturation region, the emitting OLED current can be calculated using the following equation:

(1) $$\begin{array}{ll} I_{OLED} & =\frac{1}{2}\mu_{n}C_{ox}{\left(\frac{W}{L}\right)}_{T1}{\left(V_{GS}-V_{TH\_T1}\right)}^{2}\\ & =\frac{1}{2}\mu_{n}C_{ox}{\left(\frac{W}{L}\right)}_{T1}{\left(V_{DATA}+V_{TH\_T1}-{\Delta V}_{\mu \_T1}-V_{TH\_T1}\right)}^{2}\\ & =\frac{1}{2}\mu_{n}C_{ox}{\left(\frac{W}{L}\right)}_{T1}{\left(V_{DATA}-{\Delta V}_{\mu \_T1}\right)}^{2} \end{array}$$

Based on Equation (1), V_TH_T1_ is removed, representing that the OLED driving currents are independent of the variations in V_TH_T1_. Moreover, the proposed circuit is able to alleviate the variations in mobility via the first compensation mechanism mentioned above and also through the second compensation mechanism. Based on Equation (1), the increasing μ_n_ will result in equivalently ΔV_μ_T1_ increasing, making the OLED driving currents relatively stable. Therefore, the proposed pixel circuit compensates for variations in both threshold voltage and mobility and is also unaffected by fluctuations in power supply and V_TH_OLED_, thereby improving image uniformity. Furthermore, the driving LTPS TFT enables the proposed circuit to supply an OLED driving current with low-voltage power. As a result, the proposed pixel circuit can compensate for threshold voltage, mobility, IR-drop issue, and V_TH_OLED_ with low-voltage driving, making it suitable for use in portable displays.

## 3. Analysis of Mobility Compensation Process

**Figure 3:**
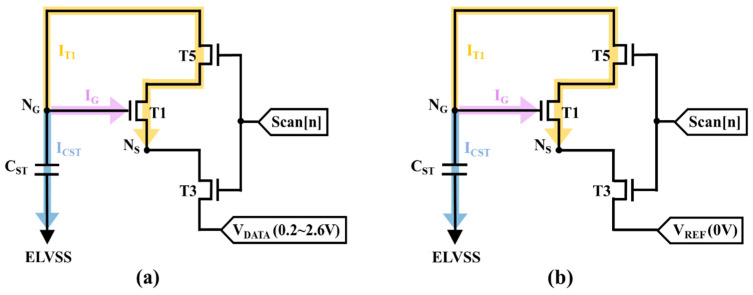
Equivalent circuit for (**a**) the programming and first mobility compensation stage and (**b**) the second mobility compensation stage for mobility compensation process analysis.

The mobility compensation feature in the proposed pixel circuit combines two different mechanisms to reduce the influence of mobility variations. To validate the process principles, the discharging process of both of the mobility compensation stages are shown in Figure 3. Figure 3a shows the equivalent circuit for the first mobility compensation stage. The transient current ($I_{CST}$) flowing through the storage capacitor can be expressed as

(2) $$I_{CST}=C_{ST}\frac{dV_{NG}}{dt}$$

where ‘$V_{NG}$’ is the voltage of N_G_ as well as the voltage being stored in storage capacitance $C_{ST}$. Subsequently, the diode-connected driving TFT discharges N_G_ from ELVDD to $V_{DATA}$ + $V_{TH\_T1}$ and the discharging current ($I_{T1}$) can be presented as

(3) $$I_{T1}=\frac{1}{2}\mu_{n}C_{ox}{\left(\frac{W}{L}\right)}_{T1}{\left(V_{NG}-V_{DATA}-V_{TH\_T1}\right)}^{2}$$

where ‘V_NG_ − V_DATA_’ represents the gate-to-source voltage ($V_{GS}$) of T1. Afterwards, the gate current of I_G_, which is much smaller than $I_{CST}$ and $I_{T1}$, can be neglected. According to the charge conservation principle, the transient current and discharging current connection can be expressed as

(4) $$I_{CST}+I_{T1}=0$$

Based on the second-order differential equations, Equation (4) can be derived as

(5) $$\frac{1}{V_{NG}{-V}_{DATA}{-V}_{{TH}_{T1}}}=\frac{1}{2}\mu_{n}C_{ox}{\left(\frac{W}{L}\right)}_{T1}\left(\frac{1}{C_{ST}}\right)\tau +A$$

where ‘τ’ is the total time for the first mobility compensation process of 7.4 μs and ‘A’ is the constant for integration. At the beginning of the operation, τ is equal to zero and V_NG_ is ELVDD; thus, the constant for the integration of A can be calculated as $\frac{1}{ELVDD{-V}_{DATA}{-V}_{TH\_T1}}$.

Therefore, the equation for V_NG_ can be presented as

(6) $$V_{NG}=\frac{1}{\frac{1}{2}\mu_{n}C_{\mathrm{OX}\;}(\frac{W}{L})T1(\frac{1}{C_{\mathrm{ST}}})\tau +\frac{1}{ELVDD{-V}_{DATA}{-V}_{TH\_T1}}}+V_{DATA}+V_{TH\_T1}$$

Equation (6) shows that as the mobility of the driving TFT increases, the voltage level of V_NG_ will be further reduced, producing less discharge current. Therefore, the correlation between the discharge current and mobility in Equation (3) can be alleviated. Because of the negative feedback system of the first mobility compensation mechanism, the fluctuation of the field-effect mobility will be relatively independent of the discharging current. In addition, the second mobility compensation mechanism is also based on the diode-connected topology, as shown in Figure 3b. The purpose of this process is to obtain a mobility-related voltage by fully discharging node N_G_ within an extremely short period. Since the node N_S_ goes to a low voltage of zero volts, the driving TFT operates in the saturation region and the discharging current (I_T1_) can be presented as

(7) $$I_{T1}=\frac{1}{2}\mu_{n}C_{ox}{\left(\frac{W}{L}\right)}_{T1}{\left(V_{NG}-V_{REF}-V_{TH\_T1}\right)}^{2}$$

Herein the transient current, gate current, and discharging current must follow the charge conservation principle shown in Equation (4). Based on the second-order differential equations, Equation (4) can be derived as

(8) $$\frac{1}{V_{NG}{-V}_{DATA}{-V}_{{TH}_{T1}}}=\frac{1}{2}\mu_{n}C_{ox}{\left(\frac{W}{L}\right)}_{T1}\left(\frac{1}{C_{ST}}\right)T+C$$

where “T” is the period for the second mobility compensation process of 0.3 μs and “C” is the constant of integration. At the beginning of the operation, T is equal to zero and V_NG_ is V_DATA_ + V_TH_T1_; thus, the value of C can be calculated as $\frac{1}{V_{DATA}{-V}_{REF}}$. After that, the voltage level of V_NG_ will be discharged to V_DATA_ + V_TH_T1_ − ΔV_μ_T1_ at the end of the compensation process. Based on the increasing field-effect mobility, discharging current multiplication will occur, which is demonstrated in Equation (7). On the other hand, ΔV_μ_T1_ can be derived as

(9) $${\Delta V}_{\mu \_T1}=\frac{1}{\frac{1}{2}\mu_{n}C_{\mathrm{OX}\;}(\frac{W}{L})T1(\frac{1}{C_{\mathrm{ST}}})T+\frac{1}{V_{DATA}{-V}_{REF}}}+V_{DATA}-V_{REF}$$

which will balance the OLED driving current variations called “the second mobility compensation mechanism”. As a result, the proposed circuit has a great capability to compensate the mobility by employing two different mechanisms.

## 4. Results and Discussion

To validate the compensation capabilities of the proposed pixel circuit, the LTPS and oxide TFTs were fabricated. The simulation parameters for AIM-Spice are listed in Table 1. Herein the simple circuit structure, which merely contains six TFTs, one storage capacitor, and five signal-controlling lines, is able to be implemented in high-resolution as well as high-PPI applications. The low voltage for V_REF_ is provided through T3 within the second mobility compensation stage to completely discharge the node N_G_. Furthermore, the controlling signal, Scan and Em, using the same voltage range, can reduce the GOA complexity and is favorable for narrow-bezel applications.

**Figure 4:**
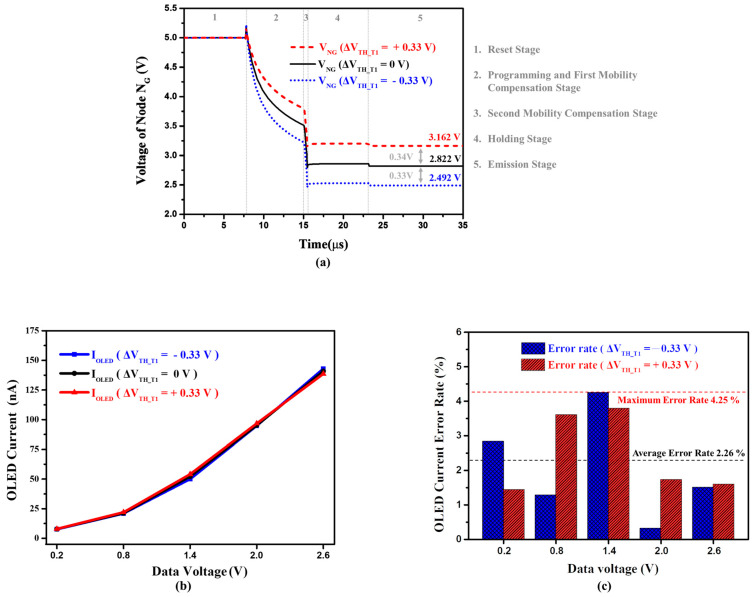
(**a**) Simulated transient waveforms of the node N_G_ voltage with an input data voltage of 2 V when V_TH_ variations in driving TFT are −0.33, 0, and +0.33 V. (**b**) Simulated OLED driving currents versus data voltage of the proposed 6T1C pixel circuit for ±0.33 V threshold voltage shifts of driving TFT, and (**c**) the corresponding currents error rate within data voltage of 0.2 to 2.6 V.

Figure 4a shows the simulated transient waveforms for node N_G_ of the proposed pixel circuit when V_TH_ variations in driving TFT are +0.33, 0, and −0.33 V, respectively. In the beginning of the operation, the voltage level of node N_G_ is reset to ELVDD (5 V), and approximately discharged to V_DATA_ + V_TH_ (3.5 V) with an input data voltage of 2 V after the programming and the first mobility compensation stage. Afterward, the V_NG_ is instantly discharged to 2.8 V by the diode-connected structure within the second mobility compensation stage. Furthermore, the proposed circuit primely senses the difference of +0.34 and −0.33 V at the end of the operation, which are nearly equal to the actual variations in ±0.33 V. Figure 4b shows the simulated OLED currents versus data voltage from 0.2 to 2.6 V for ±0.33 V threshold voltage shifts; further, the maximum current error rate of 4.25% and the average current error rate of 2.26%, which are shown in Figure 4c, demonstrate the proposed pixel circuit’s good compensation capability for threshold voltage variations.

**Figure 5:**
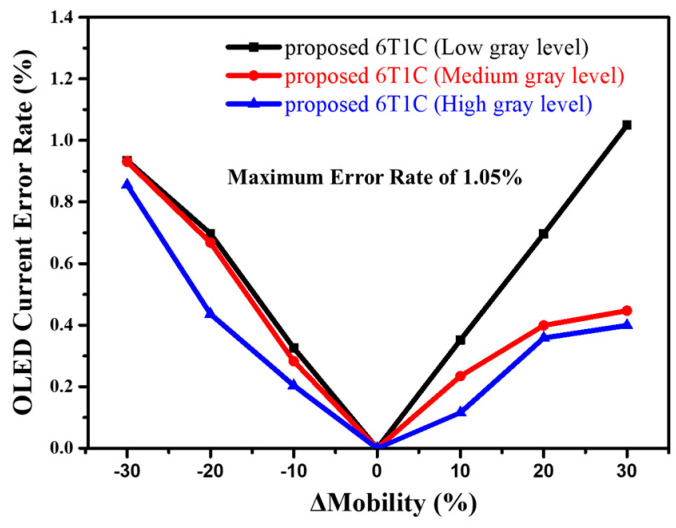
Simulated OLED current error rates at low, medium, and high gray levels when the mobility variations are ±10%, ±20%, and ±30%, respectively.

Figure 5 depicts the corresponding current error rates of the driving TFT’s mobility variations of ±10%, ±20%, and ±30%. The overall variations in the OLED driving currents are less than 1.05%, indicating that the proposed 6T1C circuit improvement not only uses the negative feedback compensation method but also adds a mobility-related parameter to significantly advance the driving TFT field-effect mobility variation immunity, and presents excellent stability when the proposed pixel circuit is operating.

**Figure 6:**
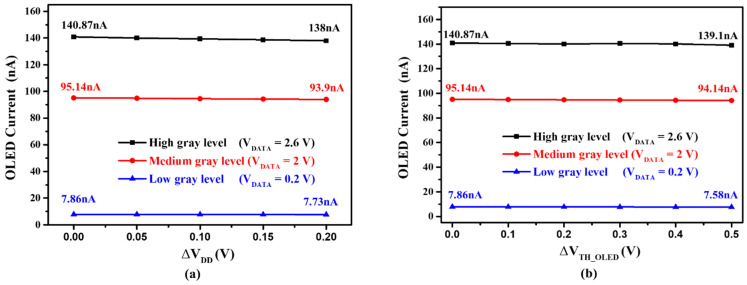
(**a**) Simulated OLED currents under the decreasing V_DD_ of 0.2 V at low, medium, and high gray levels for the proposed pixel circuit. (**b**) Simulated OLED currents under the increasing V_TH_OLED_ of 0.5 V at low, medium, and high gray levels for the proposed pixel circuit.

Figure 6a shows the OLED currents with low, medium, and high gray levels when the ELVDD voltage level drops by 0.2 V, as caused by the IR-drop issue. Herein the maximum shift at high gray level is approximately 2.07%, validating a good stability as power drops. Figure 6b shows the OLED currents with different gray levels when the V_TH_OLED_ increased by 0.5 V, implying OLED degradation. Herein the maximum shift at low gray level is about 3.61%, indicating that the proposed circuit can reduce the OLED degradation impact.

In Table 2, the comparison between the proposed and previously published pixel circuits [20,21,24,25,26] demonstrates the advantages of the proposed 6T1C circuit, including the single storage capacitor structure, the minimum numbers of signal lines, excellent V_TH_TFT_ and mobility compensation capability, independence from IR-drop and OLED degradation (V_TH_OLED_) issues, and low-voltage driving. As a result, the simulated results confirm that the proposed 6T1C pixel circuit shows a superior performance when operating under V_TH_ and mobility compensation, making it highly suitable for portable displays.

**Figure 7:**
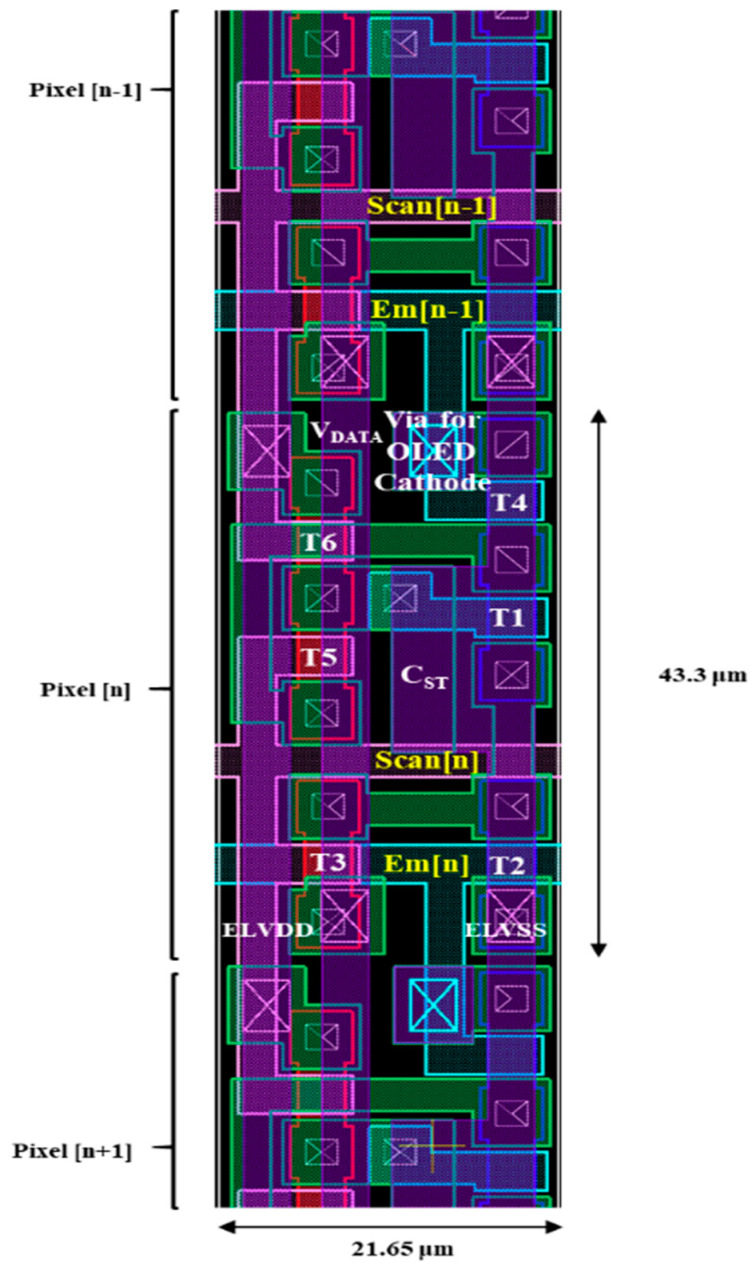
The layout of the proposed 6T1C pixel circuit.

The layout design of the proposed 6T1C circuit with signal line-sharing technology is shown in Figure 7. The display specifications of 4K (UHD) resolution and a high PPI of 587, are suitable for portable applications. With the small subpixel dimensions of 43.3 µm × 21.65 µm, it is believed to reach high pixel density for portable applications.

## 5. Conclusions

This manuscript proposed a LTPO 6T1C pixel circuit with novel operation method for AMOLED portable displays. To achieve the 4K high-resolution and high-PPI applications, the proposed pixel circuit utilizes a single storage capacitor that shares switch-controlling signals, minimizing the circuit complexity. The threshold voltage and mobility compensation capabilities are improved by both compensation mechanisms, which are based on a negative feedback system, and mobility-related compensation parameters. According to the calculation and analysis results, the mobility compensation capabilities of the proposed circuit are confirmed. Further simulation results show that the maximum error rate is 4.25% with ±0.33 V TFT threshold voltage shifts and 1.05% with ±30% mobility variations, indicating good compensation results. The proposed circuit can be driven with a low power voltage of 5 V and is also free of the IR-drop and OLED degradation problems. As a result, the proposed pixel circuit is advantageous for portable AMOLED displays.

## Figures and Tables

**Table 1:** Specifications of proposed 6T1C pixel circuit.

Proposed Pixel Circuit (6T1C)			
Parameter	Value	Parameter	Value
LTPS TFT: (W/L)_T1, T2, T4_ (μm)	3/3	ELVDD (V)	5
Oxide TFT: (W/L)_T3, T5, T6_ (μm)	3/3	ELVSS (V)	0
C_ST_ (pF)	0.1	Scan, Em (V)	−1~8
V_REF_ (V)	0	V_DATA_ (V)	0.2~2.6

**Table 2:** Comparison Between Proposed and Previously Pixel Circuits.

Reference	This Study 6T1C	Ref [20] 4T2C	Ref [21] 3T1C	Ref [24] 7T1C	Ref [25] 4T1C	Ref [26] 5T2C
Signal lines	5	7	6	6	6	6
Resolution	3840 × 2160	7680 × 4320	N/A	1920 × 1080	1024 × 768	3840 × 2160
V_TH_DTFT_ Error Rate (%)	4.25% (±0.33 V)	4.42% (±2 V)	33% (±0.6 V)	3% (±0.3 V)	9% (±2 V)	4.5% (±0.3 V)
Mobility Error Rate (%)	1.05% (±30%)	4.48% (±10%)	15% (±66%)	3% (±4.2%)	10% (±50%)	2% (±25%)
V_DD_ Compensation	O	O	O	X	O	O
V_TH_OLED_ Compensation	O	O	X	O	X	O
Power (V_DD_ − V_SS_)	5 V	20 V	12 V	7 V	10 V	11 V
